# Associations between Psycho-Hedonic Responses to Sweet and Savoury Tastes with Diet and Body Composition in a Sample of Asian Females

**DOI:** 10.3390/foods9091318

**Published:** 2020-09-18

**Authors:** Amanda JiaYing Lim, Pey Sze Teo, Vicki Wei Kee Tan, Ciarán G. Forde

**Affiliations:** 1 Clinical Nutrition Research Centre (CNRC), Singapore Institute for Food and Biotechnology Innovation (SIFBI), Agency for Science, Technology and Research (A*STAR), Singapore 117599, Singapore; Amanda_Lim@sifbi.a-star.edu.sg (A.J.L.); Teo_Pey_Sze@sifbi.a-star.edu.sg (P.S.T.); vicki_tan@sifbi.a-star.edu.sg (V.W.K.T.); 2 Department of Physiology, Yong Loo Lin School of Medicine, National University of Singapore, Singapore 117593, Singapore

**Keywords:** taste preference, adiposity, energy intake, taste cluster, dietary behaviour

## Abstract

Taste preferences guide food choices and dietary behaviours, yet few studies have shown a relationship between sweet and savoury taste preference and differences in dietary intakes or energy consumed from different “taste clusters”. We investigated differences in psycho-hedonic responses to sweet and savoury tastes and their association with energy intake, proportion of energy from macronutrients and energy intake from different “taste clusters”. In addition, we evaluated correspondence between two methods to classify “sweet-liker” status and the overlap between sweet and savoury taste preferences. Psycho-hedonic responses to sweet and savoury tastes of female participants (*n* = 66) were captured via staircase paired preference and the “sweet-liker phenotype” classification method. Quantitative dietary energy and macronutrient intakes were measured using three-day food diary, and the relative contributions of specific taste clusters to energy intake were derived for each participant. All participants completed anthropometric assessments measuring body mass index (BMI) and adiposity. Results showed no association between sweet and savoury preferences with dietary energy or macronutrient intakes, though there was a trend towards higher sweet food consumption among “sweet-likers”. A higher preference for savouriness was not associated with differences in daily energy intake, energy intake from protein, BMI or adiposity levels. There was little overlap in sweet and savoury preferences, suggesting a bi-modal split in taste preferences. “Sweet-likers” preferred a higher mean sucrose concentration than sweet “dislikers” (*p* < 0.001) indicating agreement between the two approaches. Future studies should consider comparing taste-liker differences using food choice tasks to address the current gap between taste preference measures and actual dietary behaviours.

## 1. Introduction

Food choices and intake behaviours are guided by our preferences, and these preferences are thought to inform our habitual dietary behaviour [1]. We eat what we like and is familiar and tend to avoid the tastes we dislike. The relationship between taste preferences and our dietary intake and body composition have been studied over many years, yet few studies to date have successfully confirmed a relationship between taste preference and dietary habits. Taste preferences can be objectively determined using psycho-hedonic methods where best estimate concentrations are derived using a paired preference staircase method in which preferred concentrations of a given tastant are chosen from a series of concentrations [2]. Psycho-hedonic taste profiles can differ widely by age and vary across different taste qualities but have also been shown to be stable at an individual level over time, providing insights on how liking may influence wider food choice and intake behaviour [3,4,5]. Variations in taste preferences between individuals may therefore inform differences in habitual energy intake and dietary behaviours and, over time, differences in body composition.

The five basic tastes do not contribute equally to habitual dietary energy intakes, with most energy coming from sweet, salty or savoury tasting foods and a much smaller contribution from sour or bitter foods [6]. Previous research has shown that almost 70% of daily energy intake in an Asian population is from sweet and savoury tasting foods [2,7]. Sweet and savoury taste transduction overlaps via gustatory G-coupled protein receptor systems on the tongue [8], yet to date, there is no evidence to suggest an overlap in sweet and savoury preferences, or likers for each taste consuming foods with these tastes preferentially. As the overlap between sweet and savoury preferences within the same individuals has not been explored previously, it remains unclear the degree to which liking a sweet and savoury taste overlaps and contributes to variability in dietary energy intake.

Having a heightened preference for a given taste may be associated with increased dietary intake of foods with that taste, such that individuals who prefer sweeter tastes may consume more sweet tasting foods. Previous research has shown that individuals with a higher liking for sweetness consume more energy, carbohydrates and refined sugars than those that do not have the same preference for higher sweetness [2,9]. Early work by Pangborn and Giovanni demonstrated a small but significant association between the frequency of sweet food consumption and the most preferred sucrose concentration in lemonade, in what was described as a “sweet tooth” phenotype [10]. However, others have failed to show a relationship between a higher preference for sweetness and increased dietary energy and sugar consumption [11,12], or a relationship between preferred sweetness intensity and preference across different body mass index (BMI) weight classes [13]. Findings suggest people with obesity are less sensitive to sweet taste and have increased sweet taste preference compared to their normal-weight counterparts [14]. A recent study developed a standardised “sweet liker phenotype” approach to categorise sweet-likers and found no significant difference in BMI across high and low sweet-liker phenotypes [13]. Empirical data on taste liking, diet and body composition is limited, and different approaches have been used to classify sweet likers, making it difficult to draw a comparison between studies. It remains unclear whether an increased preference for “sweetness” leads to increased sugar and caloric intake or contributes to weight gain over time.

Less is understood about the relationship between savoury (“umami”) psycho-hedonic responses and dietary intakes of savoury foods or body composition. Women with obesity were reported to prefer higher concentrations of monosodium glutamate (MSG) than those in the normal BMI range [15]. Similarly, a higher intake of MSG has previously been shown to be positively associated with higher BMI among Chinese adults [16]. Previous research has shown that dietary protein is positively correlated to the savoury intensity in foods [17], with others suggesting that low levels of dietary protein may promote a heightened preference for savoury foods and protein sources in the diet [18]. However, others have demonstrated that perceived savouriness is not a good predictor of a foods protein content [19]. Hence, the influence of savoury preferences on dietary energy and macronutrient intakes remains unclear.

Dietary intake behaviours are often compared by the relative contribution of different macronutrients to energy intakes, but it is also possible to compare intake behaviour by the predominant taste qualities of the foods consumed [17,20]. Objective profiling of food taste qualities in the diet relies on trained panel assessments to objectively score the taste predominant intensities of a representative set of foods for the five basic tastes (sweet, sour, bitter, salt and savoury) and fat sensation [21,22]. Findings from dietary taste patterns highlight that taste qualities positively associate with their respective nutrients in the Malaysian and Dutch diets, such that sweet tasting foods were higher in mono- and di-saccharides and savoury tasting foods were higher in protein [23]. Consumption of foods from different dietary taste clusters was also related to an individual’s weight status, highlighting that certain taste clusters may contribute differentially to energy intakes across weight categories [24]. Using this approach, it is possible to gather novel insights on how food intake behaviour is influenced by sensory preferences for specific tastes and apply this to explore differences in dietary patterns across taste qualities among healthy and unhealthy diets. To date the association between an individual’s psycho-hedonic taste preference for sweet and savoury tastes and the relative contribution of these taste properties to their daily energy intake has not been explored.

Whereas previous studies have explored the relationship between taste liking and body composition [12], few have considered the relationship between taste preference and differences in dietary intakes and the different taste profiles of diets. The current study aimed to (i) examine whether differences in sweet and savoury psycho-hedonic taste preferences are associated with differences in dietary energy intakes. We also sought to (ii) examine whether differences in sweet and savoury psycho-hedonic taste preferences are associated with differences in the proportion of energy from protein, fat carbohydrates and total sugar in the diet. By classifying participants as either sweet or savoury likers/dislikers, we tested (iii) whether taste liking was associated with differences in the relative consumption of energy from “taste clusters” in the diet or (iv) differed in their BMI and adiposity levels.

We hypothesised that individuals with a higher preference for sweetness would have higher energy intake and consume a higher proportion of their energy from sugars, and a higher proportion of their energy from the “sweet taste” cluster. We further hypothesised that sweet likers would have a higher BMI and adiposity compared to those preferring lower sweetness. Similarly, we hypothesised that a higher savoury preference would be associated with a higher proportion of energy from protein, and higher energy intake from the “savoury taste cluster”. Finally, we investigated the correspondence between two methods used to categorise sweet-liker phenotypes, and explored the overlap between “sweet” and “savoury” preferences within the same individuals.

## 2. Materials and Methods

### 2.1. Experimental Overview

To examine the association between psycho-hedonic preferences for sweet and savoury tastes with dietary energy intakes, participants first had their sweet and savoury taste preferences measured using a standardised staircase paired preference approach [15]. Dietary intakes were recorded by each participant using a three-day food diary (3DFD) that was coded post-hoc to derive estimates of energy and nutrient intakes. The proportion (%) of energy intake from different “taste clusters” in each individual was calculated by combining the dietary energy intake data from the 3DFD with the taste qualities data from a previously published food taste database (N_foods_ = 892) [25]. Participant body composition was measured using anthropometric and bioelectrical impedance analysis (BIA) and compared by their taste preference designation for sweet and savoury taste.

The correspondence between the sweet-liker phenotype classification method and staircase paired preference method was compared using participant responses in each method. Lastly, we explored the overlap between sweet and savoury taste preferences within the same individuals by comparing their preferred concentrations for sweet and savoury from the staircase paired preference method.

### 2.2. Sample Size Calculation

Previous research has concluded that it is not possible to date to determine the effect sizes of taste hedonics on dietary intakes or body composition due to the lack of existing evidence [2]. Given the heterogeneity of findings to date on the relationship between taste preference and dietary intakes, we have assumed an effect size of 0.875 as sufficient to detect a change in percentage of body fat (% body fat) between sweet-liker status. The power-calculation estimated a minimum total of 44 participants (22 per group) were required based on the sensitivity of taste preference measures to detect differences in % body fat, using an effect size of 0.875 with power of 80% and alpha = 0.05. The power calculation was completed using G*Power version 3.1.9.2 (Heinrich-Heine-Universität Düsseldorf, Düsseldorf, Germany)

### 2.3. Study Participants

Female participants (*n* = 69) aged between 21–50 years old were recruited to investigate the association between sweet and savoury taste preferences and energy intake, proportion of energy from macronutrients, “taste clusters” and body composition. The current study recruited only females based on previous findings that showed differences in savoury sensitivity and psycho-hedonic functions among women with obesity [15]. To explore these findings further and extend the comparison to sweet psycho-hedonic preferences, the current study explored the relationship between sweet and savoury preferences as they related to dietary intake and body composition differences among Asian females. In the final analysis, three participants were excluded due to incomplete data, resulting in a total of 66 participants in the final model. Eligible participants with no food allergies, intolerances, dietary restrictions, sinus problems or major chronic diseases were screened, and all measures were conducted in the sensory laboratory of Clinical Nutrition Research Centre. Written consent was obtained from all participants, and ethics were approved by Domain Specific Review Board of the National Healthcare Group, Singapore (DSRB 2018/01070).

### 2.4. Taste Preference Assessment

#### 2.4.1. Staircase Paired Preference Method—Sweetness and Savouriness Preferences

The staircase paired preference method was used to estimate preferred concentrations for sucrose (“sweet”) and monosodium glutamate in vegetable broth (“savoury”) based on a previously published approach [15]. Participants were required to complete a paired preference task for each sweet and savoury taste separately and were presented with pairs of 5 mL samples of two adjacent concentrations for each taste across five concentrations to identify their preferred concentration. Participants were instructed to cleanse their palette with water during an enforced one-minute break between sample pairs. For each paired preference comparison, participants chose their preferred concentration and the subsequent pairing was chosen as the first selected sample paired with adjacent concentration. Each step was repeated until participants chose the same concentration when presented with both higher and lower adjacent concentrations, or when they chose the highest or lowest concentration twice consecutively [15]. Taste preference measures were repeated in duplicate in ascending and descending series of concentrations for each taste solution. The two basic taste stimuli used were sucrose (NTUC Fair Price, Singapore) dosed in filtered water served at room temperature. For savoury taste, monosodium glutamate (MSG, Ajinomoto^®^) was added to a commercial vegetable broth (Pacific Organic Vegetable Broth) served at 40 ± 2 °C [15]. Five concentrations of 3%, 6%, 12%, 24% and 36% *w/v* sucrose were used for sweetness, while concentrations of 0.09%, 0.19%, 0.36%, 0.63% and 1.08% *w/v* MSG were used for savouriness.

#### 2.4.2. Sweet-Liker Phenotypes Classification Method

In addition to the staircase paired preference taste assessment, all participants were characterised for their sweet-liker phenotype using the sweet-liker classification method described in Iatridi, Hayes and Yeomans [13]. Three different sucrose concentrations (i.e., water (control), 0.5 M (17% *w*/*v*) and 1.0 M (34% *w*/*v*)) were presented to participants in a sequential monadic order. Participants were instructed to evaluate their liking for each solution separately on a 100 point visual analogue scale (VAS) from “Dislike extremely” to “Like extremely” [13]. Responses were used to categorise participants as “sweet likers”. For each sample evaluation, participants were asked to place the 5ml sample in their mouth and swirl for five seconds before expectoration. Measures were collected in duplicate, and sample presentation was randomised to reduce potential response bias. All data were collected using computerised data acquisition software (Compusense Cloud, Guelph, ON, Canada).

### 2.5. Dietary Assessment

To assess dietary intake, participants completed a three-day food diary on two weekdays and one weekend day [25]. The three-day dietary record has been widely used for dietary assessment and is recognised as a valid and reliable approach in providing valid estimates of dietary energy and almost all nutrient intakes [26,27,28]. A sample of a recorded day of food diary was provided and explained to participants. All completed food records were keyed into Food-Works 10 software (Food-Works Professional, Xyris Software Pty Ltd., Brisbane, Queensland, Australia) to estimate participants’ dietary energy and nutrient (carbohydrates, protein, total fat, total sugar) intakes using the local food composition databases. For each individual, total dietary energy intake and their proportion (%) of energy intake from macronutrients (i.e., carbohydrates, fats and proteins) and total sugar were tabulated and averaged over three recall days.

#### Dietary “Taste Clusters” Method

Dietary “taste clusters” were derived using a standardised method that has been described previously [21,22]. This approach has previously been used to compare the contribution of different dietary “taste clusters” to energy and nutrient intakes in studies conducted in the United States [29], Australia [21], the Netherlands [17,23], France [30] and Malaysia [7]. This approach has been applied to better understand the proportional contribution of different taste qualities to energy and nutrient intakes across food environments and relate this to differences in body compositions [24].

The approach utilised a trained sensory panel to profile the predominant taste qualities across a wide range of different foods and beverages to objectively score the taste intensities of a representative set of foods across the five basic tastes and fat sensation [21,22]. Using data from the published taste database of 892 foods and beverages from the Netherlands and Malaysia, foods were re-coded to detail the single meal ingredients for each dish. This was applied to most of the home-prepared meals and mixed dishes. For instance, “wholemeal bread” and “kaya” were re-coded as a dish “Jam, Seri kaya, with bread”. Foods responsible for ~99% of the energy intake in the current study population were classified into one of the five dietary taste clusters (“sweet-fatty”, “savour-fatty”, “sweet-sour”, “neutral” and “bitter”) based on the trained panel taste database of Malaysian and Dutch foods [22]. For foods not found in the databases, they were assigned a taste cluster based on similar foods from within the same food group, taking into account the food’s nutrient and energy contents. For example, blueberries were classified as “sweet sour” due to their similar nutrient and energy content as the strawberries in the same food group. Several supplements and condiments such as collagen powder, fish oil and wasabi were excluded as they are not common components of the everyday diet and contribute to < 1% of the total energy intake.

Using this approach, it was possible to assign taste intensities to each of the foods consumed by each participant in their three-day food diary and from this derive the proportional contribution of each “taste cluster” to their energy intakes [22]. This enabled a comparison of the relative contributions of “taste clusters” to dietary energy intake and to compare this across participants with different preferences for sweet and savoury tastes as defined earlier (Section 2.4.1).

### 2.6. Anthropometric and Physical Activity Assessment

All participants had their body weight (kg, rounded to nearest 0.1 kg) and height (cm, rounded to nearest mm) measured in duplicates (Seca763 digital scale, Birmingham, UK) with light clothing on and barefooted. Body Mass Index (BMI: kg/m^2^) was derived by taking average body weight (kg) divided by the square of average height (in m^2^). The Asian BMI classification was used to categorise participants into underweight (BMI < 18.5), normal (BMI 18.5–22.9), overweight (BMI 23.0–27.4) and obese (BMI ≥ 27.5) groups [31]. Percentage of body fat was measured using an 8-electrode Bioelectrical Impedance Analyser (BIA, Tanita BC-418, Tokyo, Japan).

Participant’s physical activity level was captured using a physical activity diary, where physical activity was tracked for every 15-min block for 24 h, over seven consecutive days. A seven day period was used to minimise day-to-day variability of physical activity performed by participants [32]. For each of the 15-min blocks, participants were asked to classify their activity with reference to pre-defined categories [33]. We classified the activities according to the intensity level (low, moderate, vigorous) using the classification in Ainsworth compendium [34]. The total moderate-to-vigorous physical activity duration (min/day) was then calculated from the seven-day physical activity diary according to the intensity level of those reported activities.

### 2.7. Data Analysis

Descriptive statistics were reported as mean (SD), unless otherwise indicated. For the staircase paired preference test method, participants were split by their selections into preference for higher concentration or preference for lower concentration for both sweetness and savouriness using the respective median values of their optimum preferred concentrations for sucrose and MSG [35].

For the sweet-liker phenotypes classification method, sweet likers were categorised as individuals whose VAS ratings of liking increased with progressive increases in sucrose concentration. Sweet “dislikers” had a decrease in VAS liking ratings with increasing sucrose concentrations or rated the 0.5 M sucrose solution as most liked, in line with a previously published approach [13].

Sweet liking from the psycho-hedonic and sweet-liker phenotypes classification method were compared to investigate the correspondence between the two methods. Furthermore, the overlap between sweet and savoury preferences within the same participants was compared by plotting the preferred optimum concentration for sucrose and MSG in vegetable broth from the staircase paired preference task in a scatter plot.

The association between sweet or savoury preference with dietary energy intake and proportion of energy from macronutrients, total sugar and the relative contribution of “taste clusters” to energy intake was investigated using multivariate analysis of covariance (MANCOVA), after adjusting for age (years). Associations between sweet and savoury preferences with body composition (BMI and adiposity) was explored using MANCOVA model after adjusting for age (years) and total moderate-vigorous physical activity (mins/day). All statistical analysis was performed using SPSS software (IBM SPSS statistic; version 26, IBM, New York, NY, USA), *p* < 0.05 were considered as statistically significant different.

## 3. Results

### 3.1. Participants Characterisics

Characteristics of female participants are presented in Table 1. Mean age of the participants was 29.47 years and most participants were within the normal range of BMI (21.38 kg/m^2^) with a mean percentage body fat of 28.29%. Of 66 participants, 27% were classified as sweet likers via the sweet-liker phenotype classification method. Whilst, one-third of the participants were classified as preferring a higher concentration of sweetness and 45% classified as individuals who prefer a higher concentration of savouriness, using the staircase paired preference method.

### 3.2. Correspondence between Sweet-Liker Status and Paired Preference Optimum Concentration for Sucrose

**Figure 1:**
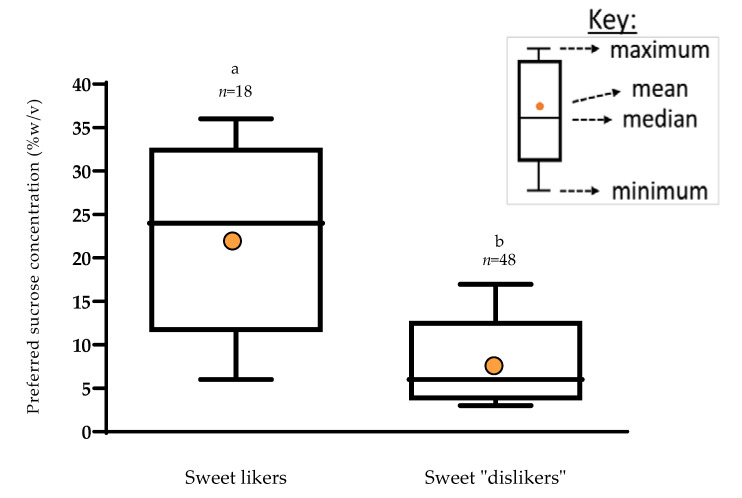
Boxplot showing preferred sucrose concentration (% *w*/*v*) by sweet-liker phenotype status (*n* = 66).

Results from the staircase paired preference and sweet-liker phenotype classification were compared within the same participants to check the correspondence between optimum sweetness concentration and sweet-liker classification method. Sweet likers preferred a significantly higher sucrose concentration of 21.6% *w/v* compared to those not classified as sweet likers (7.32% *w*/*v*) (*p* < 0.001), confirming good agreement between the sweet-liker phenotype method and a preference for higher sucrose concentrations among sweet likers (Figure 1).

### 3.3. Overlap between Sweet and Savoury Taste Preferences

**Figure 2:**
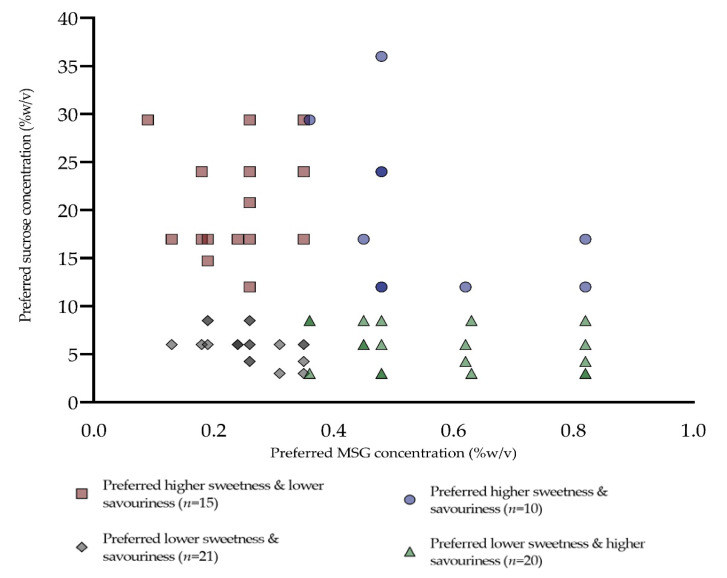
Distribution of participants’ sweetness and savouriness preferences based on optimum concentrations (% *w*/*v*) of sucrose and MSG (*n* = 66).

Figure 2 shows a scatter plot of the distribution of each participant’s preferred concentration for sucrose and MSG based on the staircase paired preference. Participants were split into four groups based on their liking for each taste. The majority of the participants preferred either a higher concentration of sucrose or a higher concentration of MSG, with only 15% of participants preferring both higher concentrations of sucrose and MSG, and 32% that preferred a lower concentration for both tastes.

### 3.4. Associations between Sweet and Savoury Taste Preferences, Energy Intake and % Energy from Macronutrients and Total Sugar

The associations between sweet and savoury preferences with energy intake and proportion of energy intake from specific macronutrients (% energy) and total sugars are summarised in Table 2. In unadjusted data, total average daily energy intake was 1740 kcal, with proportional energy intakes of 47.0% from carbohydrates, 33.6% from fat, 18.4% from protein and 9.7% from sugar.

Overall, there were no significant differences found between the participants classified by their sweetness preferences on total energy intake or % energy from total sugar after multivariable adjustment. For savoury preference, there was no difference in dietary energy intake between individuals with different preferences and no significant association between higher preference for savoury and dietary energy intake from protein. Those who preferred a higher concentration of sweetness had slightly higher average energy intake than those who preferred a lower concentration of sweetness (Δ = ↑112 kcals/day) and consumed more energy intake from sugar, although these differences were not significant. Those with a preference for higher savouriness had a lower average daily energy intake (Δ = ↓33 kcals/day) than those who preferred lower savouriness and consumed a slightly higher %energy from protein and a lower % energy from sugar, although again these differences were not significant.

### 3.5. Associations between Sweet and Savoury Taste Preferences and % Energy from “Taste Clusters” and Discretionary Foods

The associations between sweet and savoury preferences with % energy from the five different taste clusters and discretionary foods are summarised in Table 3. At the unadjusted group level, energy intakes differed by taste clusters with most energy from savoury-fatty foods (59%), followed by the neutral-taste cluster (21%), sweet-fatty (12%), sweet-sour (6%) and bitter taste cluster (2%). Among discretionary foods, half of the energy consumed was from sweet discretionary foods, compared to savoury discretionary foods.

Participants who preferred a higher concentration of sucrose consumed a higher % energy from discretionary foods, with a higher intake of sweet discretionary foods, although this difference was not statistically significant after adjusting for confounders. Those preferring a higher concentration for sweetness also consumed more energy from sweet tasting foods, from both sweet-sour and sweet-fatty taste clusters, although differences were not significant. Those preferring higher savoury taste consumed a smaller proportion of energy from total, sweet and savoury discretionary foods, with lower intake of both sweet and savoury tasting foods. A preference for higher savoury taste was significantly associated with a higher % energy intake from foods from the neutral “taste cluster” (*p* = 0.041).

### 3.6. Associations between Sweet-Liker Phenotype, Sweet and Savoury Taste Preference and Body Composition

Table 4 summarises associations between participants’ sweet-liker phenotype and sweet and savoury taste preferences with body weight, BMI and percentage of body fat after adjusting for confounders. Participants classified as sweet likers using the sweet-liker phenotypes classification method (*n* = 18) trended towards a higher percentage body fat (*d.f.* = 1, *F* = 3.25, *p* = 0.076), although the difference was not significant.

No association was observed between liking a higher sucrose concentration from the staircase paired preference method and body composition. Those who preferred a higher sweetness concentration trended towards having greater body weight, BMI and percentage body fat than those preferring a lower sweetness concentration. Participants with a higher preference for savoury taste had a higher body weight and BMI but similar percentage body fat, compared to those preferring lower savoury taste, and none of these differences were statistically significant (Table 4).

## 4. Discussion

We explored whether differences in liking for a taste was associated with differences in dietary energy intake, proportion of energy intake from macronutrients, total sugar and proportion of energy from different “taste clusters”. Findings showed no associations between sweet and savoury taste preferences and energy intake or macronutrient intakes. There was no association between sweet liking phenotype or a higher sweetness preference with body composition, although sweet likers trended towards a higher % body fat compared to those not classified as sweet likers. Moreover, there was no significant relationship between a higher preference for savoury taste and energy intakes, energy from protein or body composition. Those with a higher savoury taste preference consumed significantly more of their daily energy from the “neutral” taste cluster. The current study compared the overlap between sweet and savoury preferences within the same individuals and showed that the majority of participants preferred either sweet or savoury tastes, with only a small proportion preferring high concentrations for both tastes. Methodologically, we confirmed a good agreement between the sweet-liker phenotype classification method and the staircase paired preference method.

There was no significant association between higher sweet preference and total energy intake, proportion of energy from sugar, sweet discretionary foods or sweet tasting foods. Those preferring a higher sweetness concentration trended towards a higher intake of sweet food, though overall differences were not significant. A recent study found no significant differences in dietary intake of carbohydrates, sugars or the proportion of total energy from sugars among sweet likers, although as with our study they also showed sweet likers had higher intakes in these categories [36]. These results suggest a possible trend towards higher “sweet” intakes among likers and it may be that current approaches lack the sensitivity to detect differences in dietary intakes. A preference for higher sweetness did not track against sweet food or sugar intakes in the current study. Others have shown that in addition to preference for sweetness, differences in sweet taste sensitivity (psychophysical response and threshold sensitivity) showed no relationship with dietary intakes of sugar [12,37,38]. In a similar study, sweet likers consumed more of their energy from sugar-sweetened beverages, although differences observed were not significant [39]. A possible reason for this lack of correspondence between sweet preferences and sweet food intakes may be in the predictive validity of the psycho-hedonic measures used to characterise a higher liking for sweetness. Whereas psycho-hedonic ratings of sucrose solutions in water provide a reproducible and objective approach to characterising sweet taste preference, they do not reflect the complexity of sweet stimuli encountered in the food environment [36,40,41]. Recent findings further support this and concluded that liking for taste solutions was a poor predictor of liking for sweet tasting foods [42]. Further consideration should be given to the overall sensory properties and product context when relating sweet liking to habitual consumption of sweet foods [40].

We hypothesised that individuals that prefer a higher savoury taste intensity would consume greater energy from protein and have a higher intake of savoury discretionary foods. Those preferring higher savoury taste intensity had a slightly higher proportion of their energy intake from protein but a lower proportion of energy from savoury discretionary foods and savoury taste clusters. In all cases, the differences were not significant from those that preferred lower savoury taste. Although savoury tasting foods are common in the Asian diet, they are often not high in protein [7,43], due to the widespread use of flavourings, seasonings or usage of condiments. Previous research has highlighted that many modern processed foods can be high in savoury taste, but low in protein, suggesting that savoury taste and protein content may not be well linked [19]. Unlike carbohydrates and fat, protein intake is homeostatically regulated, and differences in protein needs have been suggested to affect preference and intake for savoury tasting foods [18]. A low protein diet or prolonged protein deprivation could enhance a preference for savoury foods as a cue to indicate higher protein content [18,44]. Others have shown that when savoury taste is paired with higher protein content, it can have a strong impact on post-meal satiety, suggesting that the congruency of taste and macronutrient pairings may play a role in post-meal satiety associated with protein [45,46].

There was no association between sweet liking or preference with body composition. Although the differences were not significant, sweet likers in the current study trended towards a higher percentage body fat than those who were not classified as sweet likers. Previous research has suggested that body fat is a more accurate estimation of adiposity than BMI in an Asian population [47,48]. Our findings are consistent with previous studies, where BMI did not differ across sweet liker phenotypes [13,39,49]. Previous research has also found no difference in sweet liking between normal weight and people with obesity [50,51,52].

Our findings showed that individuals with a preference for higher savoury taste intensity had slightly higher body weight and BMI than those preferring lower concentrations, though differences were not significant. A similar absence of a link between savoury taste preference and body mass was seen previously, where no association was found for any taste and hedonic ratings among a cohort of people with obesity [52]. By contrast, women with obesity were less sensitive to MSG and preferred higher concentrations than normal weight women [15,53]. In this regard, data are equivocal. Recent research has shown differences in how people “value” the protein content of food. Those who consistently selected foods with higher protein in a computer-based task were shown to have higher protein intake and lean muscle mass [54]. Further research is needed to understand how a food’s protein content influences dietary protein selection and intake, and how this relates to differences in savoury taste preferences.

Sweet likers reported a significantly higher preferred sucrose level (21.6% *w*/*v*) compared to those not classified as sweet likers (7.3% *w*/*v*), confirming an overlap between the two methods of “sweet-liker” classification. This is in agreement with previous findings which showed sweet likers gave higher liking ratings for the highest sucrose concentrations [13]. The proportion of sweet likers to dislikers in the current study is similar to recent findings by Iatridi and colleagues [13], where they found 31% of participants were classed as sweet likers, whereas in the current study we had 27%. The sweet-liker phenotypes classification approach has been shown to be reproducible and provides a rapid approach for categorising sweet likers [12], without the need for the more cumbersome and time-consuming staircase paired preference method. We identified four distinct groups based on their sweet and savoury taste preferences, with majority of the participants preferring either a higher sweetness or a higher savoury taste but only 15% liking both. This suggests a bi-modal split in consumer taste preferences. Dietary savoury taste is often consumed in the form of solid savoury meals, and the liquid taste solution used for the psycho-hedonic preference method may not accurately reflect the diversity of savoury experiences in the food environment. In addition to MSG, savoury taste is imparted by a wide range of ribonucleotides (i.e., inosine monophosphate/guanosine monophosphate), which are predominantly found in high protein foods such as meat and fish [19,45].

Linking taste preferences to habitual choice and dietary intake behaviours is more complicated than simply eating more of what we like. Numerous studies have failed to show any meaningful relationship between taste liking or sensitivity, and dietary intakes. In addition to homeostatic needs, many other consumption contexts, product variables and health related motivations and behaviours can influence food choice and intake behaviour. Previous research and a recent review of available data showed that exposure to sweet taste had the acute effect of reducing preference for and intake of sweetness, but had little long term effect on sweet food intake [55]. Short term effects of sweet taste exposure have been attributed to sensory-specific satiety, with limited long term effects of taste exposure on preference or intake [55,56]. The reverse is likely also true, where for example one study showed that a one-week of chocolate deprivation induced an increase in liking and desire for chocolate among regular chocolate consumers [57]. In this regard, recent dietary taste exposure may have acute effects on intake, whereas psycho-hedonic taste preferences may have a less consistent effect on intake behaviour. Future research is needed to clarify the predictive validity of taste-liker status, and associated links to food choice and dietary intakes.

Moving beyond the narrow view that liking is innate and universal to recognise individuals with distinct taste preferences could help to tailor personalised dietary advice while maintaining eating pleasure. Classifying individuals by their taste-liker status helps to better identify those with a propensity to consume greater amounts of energy from added sugar or sweet energy dense foods, who could benefit from a reduction in the sugar content of their diets. Sweet likers in the current study had a trend toward higher sugar and sweet food consumption, and with the advent of non-nutritive and low-calorie sources of sweetness, there is potential to reduce their consumption of sugars, without compromising on preferred sweetness levels. Extensive research has demonstrated that replacing sugar with low-calorie sweeteners can be used to decrease energy intake and reduce body weight [58].

The current study had several strengths; both sweet and savoury preferences were measured empirically using standardised procedures within the same population, alongside detailed information on body composition and quantitative information on energy and macronutrient intakes. However, it is important to acknowledge several sampling and methodological limitations. Despite efforts to recruit a diverse study sample, we chose to recruit female participants in the current study, and they were for the most part within the normal BMI range, which likely limited the comparisons drawn between taste preferences and BMI and adiposity. In addition, it is likely there are measurement errors for energy intakes derived from self-report dietary measures, which have previously been shown to under-report intakes [59]. Whereas we account for differences in physical activity in our analysis, we cannot rule out the possibility of residual confounding from unmeasured co-variates. The current study related taste preferences to diet, but we did not measure “food choice” using a food choice task, where sweet and savoury likers were free to select and consume preferred foods from a buffet of sweet and savoury items. Future research should consider including such real choice measures to bridge the gap between objective taste preference measures, food choice and dietary intake behaviours.

## 5. Conclusions

The current study showed no association between sweet and savoury preference and energy or macronutrient intakes. Although the results were not significant, there was a trend towards higher discretionary food, total sugar and sweet food intakes among sweet likers, and these individuals also trended towards a higher percentage of body fat than those who are not classified as sweet likers. Savoury likers tended to consume more energy from “neutral” taste clusters but with nominal differences in dietary intake and body composition. Most participants were either sweet or savoury taste likers, with a small proportion liking high intensity of both tastes. The sweet-liker phenotype classification approach was confirmed to be reproducible and associated with the psycho-hedonic staircase paired preference method. Future studies should extend these methods further to understand food choice and dietary intake behaviour within a population-based sample.

## Figures and Tables

**Table 1:** General characteristics of female participants (*n* = 66) ^1^.

Participant Characteristics	
Age, years	29.47 (8.44)
Sweet likers, *n* (%) ^2^	18 (27.27)
Sweet “dislikers”, *n* (%) ^2^	48 (72.73)
Preferred higher concentration of sweetness, *n* (%) ^3^	25 (37.88)
Preferred lower concentration of sweetness, *n* (%) ^3^	41 (62.12)
Preferred higher concentration of savouriness, *n* (%) ^3^	30 (45.45)
Preferred lower concentration of savouriness, *n* (%) ^3^	36 (54.55)
Height, (m)	1.61 (0.57)
Body weight, (kg)	56.04 (11.50)
Mean Body Mass Index, BMI, (kg/m^2^)	21.38 (4.34)
Underweight BMI < 18.5, *n* (%)	13 (19.70)
Normal weight BMI 18.5–22.9, *n* (%)	35 (53.03)
Overweight BMI 23.0–27.4, *n* (%)	13 (19.70)
Obese BMI ≥ 27.5, *n* (%)	5 (7.58)
Percentage of body fat, %	28.29 (7.42)

^1^ Unadjusted data. Values are presented as mean (SD) unless otherwise noted. Assessed by ^2^ sweet-liker phenotype classification method; ^3^ staircase paired preference method.

**Table 2:** Associations between sweetness and savouriness preferences with energy intake and proportion of energy intake (%energy) from macronutrients and total sugar (*n* = 66).

	Total ^1^(*n* = 66)	Sweetness Preference ^2^			Savouriness Preference ^2^		
		Higher (*n* = 25)	Lower (*n* = 41)	*p*-Value [95% CI]	Higher (*n* = 30)	Lower (*n* = 36)	*p*-Value [95% CI]
Energy intake (kcal/day)	1739.70 (46.80)	1809.52 (74.06)	1697.12 (57.83)	0.236 [−75.38, 300.18]	1721.46 (68.32)	1754.89 (62.36)	0.719 [−218.31, 151.44]
**% energy**							
Carbohydrates	46.99 (0.89)	47.17 (1.41)	46.88 (1.11)	0.872 [−3.30, 3.88]	47.34 (1.29)	46.70 (1.18)	0.713 [−2.85, 4.14]
Fat	33.63 (0.74)	33.89 (1.18)	33.47 (0.92)	0.777 [−2.56, 3.41]	33.38 (1.08)	33.84 (0.98)	0.753 [−3.37, 2.45]
Protein	18.41 (0.60)	18.08 (0.97)	18.61 (0.76)	0.673 [−2.99, 1.94]	18.53 (0.89)	18.30 (0.81)	0.851 [−2.18, 2.63]
Total Sugar	9.68 (0.61)	10.27 (0.99)	9.31 (0.77)	0.451 [−1.56, 3.47]	8.41 (0.88)	10.73 (0.81)	0.057 [−4.72, 0.07]

^1^ Unadjusted data. Values are presented as mean (SE). ^2^ Adjusting for age (years). Values are presented as mean (SE).

**Table 3:** Associations between sweetness and savouriness preferences with proportion of energy intake (% energy) from taste clusters and discretionary foods (*n* = 66).

% Energy	Total ^1^ (*n* = 66)	Sweetness Preference ^2^			Savouriness Preference ^2^		
		Higher (*n* = 25)	Lower (*n* = 41)	*p*-Value [95% CI]	Higher (*n* = 30)	Lower (*n* = 36)	*p*-Value [95% CI]
Savoury fatty taste cluster	58.57 (1.83)	56.87 (2.93)	59.61 (2.29)	0.463	56.75 (2.67)	60.09 (2.43)	0.359
				[−10.16, 4.68]			[−10.55, 3.88]
Neutral taste cluster	20.55 (1.48)	20.39 (2.39)	20.65 (1.86)	0.932	23.79 (2.11)	17.84 (1.92)	0.041 *
				[−6.31, 5.79]			[0.25, 11.66]
Sweet fatty taste cluster	12.46 (1.17)	13.76 (1.89)	11.66 (1.48)	0.384	12.19 (1.74)	12.68 (1.59)	0.835
				[−2.70, 6.91]			[−5.20, 4.22]
Sweet sour taste cluster	6.05 (0.69)	6.15 (1.10)	5.99 (0.86)	0.907	4.69 (0.98)	7.18 (0.90)	0.066
				[−2.63, 2.96]			[−5.14, 0.17]
Bitter taste cluster	2.15 (0.45)	2.66 (0.72)	183 (0.56)	0.369	2.30 (0.66)	2.02 (0.60)	0.755
				[−1.00, 2.65]			[−1.50, 2.07]
Total discretionary foods	31.41 (1.77)	33.38 (2.78)	30.21 (2.17)	0.371	30.37 (2.55)	32.28 (2.32)	0.583
				[−3.86, 10.22]			[−8.79, 4.98]
Sweet discretionary foods	17.96 (1.30)	19.56 (2.12)	16.99 (1.66)	0.345	16.27 (1.93)	19.38 (1.76)	0.240
				[−2.82, 7.95]			[−8.33, 2.12]
Savoury discretionary foods	4.80 (0.65)	5.05 (1.07)	4.65 (0.84)	0.769	4.12 (0.97)	5.37 (0.89)	0.346
				[−2.31, 3.11]			[−3.87, 1.38]

^1^ Unadjusted data. Values are presented as mean (SE). ^2^ Adjusting for age (years). Values are presented as mean (SE). * Significant difference at *p* < 0.05.

**Table 4:** Associations between sweet-liker phenotypes, sweetness and savouriness preferences and body composition profiles (*n* = 66) ^1^.

	Sweet-Liker Phenotypes			Staircase Paired Preference Method					
				Sweetness Preference			Savouriness Preference		
	Sweet Likers (*n* = 18)	Sweet “Dislikers” (*n* = 48)	*p*-Value [95% CI]	Higher (*n* = 25)	Lower (*n* = 41)	*p*-Value [95% CI]	Higher (*n* = 30)	Lower (*n* = 36)	*p*-Value [95% CI]
Body weight, kg	58.22 (2.56)	55.22	0.322	57.86 (2.15)	54.93 (1.68)	0.287	56.94 (1.98)	55.29 (1.81)	0.541
		(1.56)	[−0.38, 7.31]			[−2.53, 8.39]			[−3.72, 7.02]
Body Mass Index, BMI, kg/m^2^	22.56 (0.95)	20.94	0.150	22.07 (0.81)	20.96 (0.63)	0.279	21.63 (0.74)	21.17 (0.68)	0.650
		(0.58)	[−0.61, 3.85]			[−0.93, 3.16]			[−2.47, 1.55]
Percentage body fat, %	30.81 (1.64)	27.34	0.076	29.52 (1.40)	27.54 (1.09)	0.270	28.19 (1.30)	28.37 (1.18)	0.916
		(1.00)	[−3.01, 9.00]			[−1.58, 5.53]			[−3.70, 3.33]

^1^ Adjusting for age (years), total moderate-to-vigorous physical activity (min/day). Values are presented as mean (SE).
